# Transcriptomic Analysis of Acetaminophen Biodegradation by *Penicillium chrysogenum* var. *halophenolicum* and Insights into Energy and Stress Response Pathways

**DOI:** 10.3390/jof9040408

**Published:** 2023-03-27

**Authors:** Francisco J. Enguita, Sofia Pereira, Ana Lúcia Leitão

**Affiliations:** 1 Instituto de Medicina Molecular João Lobo Antunes, Faculdade de Medicina, Universidade de Lisboa, Av. Professor Egas Moniz, 1649-028 Lisboa, Portugal; 2 Department of Chemistry, NOVA School of Science and Technology, FCT NOVA, Universidade NOVA de Lisboa, 2829-516 Caparica, Portugal; 3 MEtRICs, NOVA School of Science and Technology, FCT NOVA, Universidade NOVA de Lisboa, 2829-516 Caparica, Portugal

**Keywords:** acetaminophen, biodegradation, *Penicillium chrysogenum*, transcriptomic analysis, functional networks

## Abstract

(1) Background: Acetaminophen (APAP), an active component of many analgesic and antipyretic drugs, is one of the most concerning trace contaminants in the environment and is considered as an emergent pollutant of marine and aquatic ecosystems. Despite its biodegradability, APAP has become a recalcitrant compound due to the growth of the global population, the ease of availability, and the inefficient wastewater treatment applied. (2) Methods: In this study, we used a transcriptomic approach to obtain functional and metabolic insights about the metabolization of APAP by a phenol-degrading fungal strain, *Penicillium chrysogenum* var. *halophenolicum*. (3) Results: We determined that the transcriptomic profile exhibited by the fungal strain during APAP degradation was very dynamic, being characterized by an abundance of dysregulated transcripts which were proportional to the drug metabolization. Using a systems biology approach, we also inferred the protein functional interaction networks that could be related to APAP degradation. We proposed the involvement of intracellular and extracellular enzymes, such as amidases, cytochrome P450, laccases, and extradiol-dioxygenases, among others. (4) Conclusions: Our data suggested that the fungus could metabolize APAP via a complex metabolic pathway, generating nontoxic metabolites, which demonstrated its potential in the bioremediation of this drug.

## 1. Introduction

The chemical structure of Acetaminophen (APAP), also known as paracetamol, N-4-hydroxyphenyl)ethanamide, N-acetyl-p-aminophenol, 4-acetaminophenol, or 4-hydroxyacetanilide, contains a benzene ring core substituted by one hydroxyl group and the nitrogen atom of an amide group in the *para* position. In 1948, Brodie and Axelrod discovered that the analgesic effect of acetanilide was due to its active metabolite APAP, and since then, it has been widely used as a pain reliever and antipyretic [1]. While it is generally safe to take it at a low dosage (the FDA-approved maximum dose is less than 4 g per day), APAP is a major cause of acute liver failure worldwide [2,3].

Due to its high consumption and emission rates from manufacturing facilities and hospitals, APAP is continuously introduced into aquatic environments, with it being considered as an emerging pollutant. Despite wastewater treatment plants being the main source of APAP into the environment [4,5,6], it has been detected in several rivers, lakes, and groundwater reservoirs. Individual studies performed in surface waters from Malaysia [7], Spain [8,9], China [4], and Portugal [10] allowed determining the presence of APAP in a wide range of concentrations. Moreover, a review article aiming to compare African with European pharmaceuticals in freshwater aquatic environments concluded that the maximum concentration of APAP in African aquatic environments is approximately 215 times higher than the concentrations reported in European studies [11].

Based on a literature survey and an extensive set of criteria such as regulation, consumption/sales, physicochemical properties, toxicity/ecotoxicity, occurrence in surface and drinking waters, groundwater and wastewater, as well as degradability/persistence and resistance to treatment, APAP was considered in the class 2 as priority pharmaceuticals [12]. Ashfaq and coworkers estimated the ecological risks in wastewater and the results indicated a medium risk of APAP against green algae and a high risk against *Scinax proboscideus* and *Daphnia magna* [13]. Recently, Lindim and coworkers predicted the ecotoxicological impact resulting from the exposure of aquatic organisms to the mixture of pharmaceuticals in Swedish freshwaters and concluded that APAP, among four other pharmaceuticals (namely diclofenac, ethinylestradiol, erythromycin, and ciprofloxacin), are associated with chronic risks [14].

APAP is mainly removed from wastewaters using chemical processes such as ozonation, electrochemical, TiO_2_, and Fenton oxidation [15,16,17,18]. Despite the efforts in the optimization of methodologies that allow eliminating APAP, the current methods are still complicated, cost-prohibitive, and present environmental constraints [19,20,21]. So, APAP is a current and future threat to ecosystem homeostasis due to the growing global population, considering its presence as the main active ingredient in many prescriptions, the ease of availability, and inefficient wastewater treatment processes. Bioremediation is an ecofriendly, noninvasive, and cheaper alternative to conventional methods, since it is a permanent solution that can end with the transformation of environmental pollutants into harmless compounds such as CO_2_ and H_2_O, or less toxic forms [22]. However, APAP biodegradation could create products that are equal pollutants and could even represent a higher threat to human health due to their potential as mutagenic and carcinogenic compounds. Examples of them are hydroquinone and p-aminophenol, which have been reported as products of APAP biodegradation [23,24]. In 2018, Zur et al. compiled the data on and advances in APAP degradation by bacteria, giving insights on the biodegradation mechanisms for APAP detoxification [25]. Recently, Rios-Miguel et al. also highlighted the large diversity of bacterial amidases involved in APAP metabolism [26].

In this field, mycoremediation could make an important contribution due to the promiscuous nature of fungal enzymes and their ability to adapt to adverse environmental conditions. *Penicillium* spp. have been used in the remediation of organic compounds and heavy metals [27]. Interestingly, despite the few reports of APAP mycoremediation [28,29,30,31], *Penicillium* was the first fungal species described to be able to degrade APAP [30]. In our lab, we isolated and characterized a *Penicillium* strain designated as *P. chrysogenum* var. *halophenolicum* (previously known as *Penicillium chrysogenum* CLONA2) [32]. Despite *Penicillium chrysogenum* being reclassified as *Penicillium rubens* [33], we decide to maintain the name as *P. chrysogenum* var. *halophenolicum* in order to avoid confusion with the previously published work. This fungal strain was used before for the remediation of hydroquinone and catechol among other phenolic compounds without previous acclimation under minimal nutritional requirements [34,35]. In this work, we investigated the APAP degradation ability of the selected fungal strain and characterized the transcriptomic response of the fungus against APAP, giving some insights related to the possible pathways involved in the metabolization of the drug. 

## 2. Materials and Methods

### 2.1. Chemicals

Acetaminophen (APAP), as well as catechol (CAT), hydroquinone (HQ), and p-aminophenol (APA), were purchased from Sigma-Aldrich (St. Louis, MO, USA). All other reagents were of analytical reagent grade and were obtained from Riedel-de Haën (Seelze, Germany). Water purified with a Mili-Q system was used in all the experiments. 

### 2.2. Microorganisms and Culture Conditions

*P. chrysogenum* var. *halophenolicum*, which was isolated from a salt mine in the Algarve region, Southern Portugal and previously characterized [32, 36], was used in this study. The fungal strain was grown on solid Power medium [37] for 6 days at 25 °C and was maintained at 4 °C for further experiment use. 

Fungal precultures were obtained from conidia grown on Power medium and cultivated in 300 mL flasks containing 80 mL of modified MC medium (per liter: 30 g of glucose, 3.0 g of NaNO_3_, 0.50 g of MgSO_4_·7H_2_O, 10 mg of NH_4_Fe(SO_4_)_2_·12H_2_O, 1.0 g of K_2_HPO_4_, 5.0 g of yeast extract, and 20 g of NaCl; the pH was adjusted to 5.6–5.8 with 5.0 M HCl) at 160 rpm in an orbital shaker (Certomat^®^ BS-T Incubator, Sartorius Stedim Biotech, Gottingen, Germany) at 25 ± 1 °C for 68 h. The cells were centrifuged for 10 min at 10,000× g and washed three times in 0.85% (*w*/*v*) of NaCl. Then, a 10% aliquot constituted the biomass of the fungal precultures. 

### 2.3. Acetaminophen Removal Experiments

*P. chrysogenum* var. *halophenolicum* cultures were performed by inoculating the biomass from precultures on 50 mL of defined minimal medium (per liter: 1.0 g of K_2_HPO_4_, 3.0 g of (NH_4_)_2_SO_4_, 200 mg of MgSO_4_·7H_2_O, 33 mg of FeCl_3_·6H_2_O, 100 mg of CaCl_2_, and 20 g of NaCl; pH was adjusted to 5.6–5.8 with 5.0 M HCl) supplemented with acetaminophen (APAP) and/or glucose (1.5 mg/mL) in 250 mL flasks and incubated at 25 °C in an orbital shaker (160 rpm). An APAP concentration of 2.00 mM was used in all the experiments, except in the case of removal ability experiments, where concentrations of 0.661, 1.00, 2.00, 3.00, 4.00, and 5.00 mM were tested. The experiments were performed in triplicates. Uninoculated control flasks (duplicates) were incubated and aerated in parallel as negative controls.

### 2.4. Analytical Methods

Microbial dry biomass was estimated gravimetrically in duplicate. Each sample of 50 mL was filtered through quantitative filter paper (VWR International, Leuven, The Netherlands). The fungal pellets were dried at 60 °C for 48 h. The samples were placed in a desiccator for 60 min and weighted afterwards. The concentration of biomass was calculated as grams of dry biomass per liter of medium. To determine glucose consumption in fungal cultures, the glucose concentration was measured using the commercial kit D-Glucose GOD-POD (NzyTech, Lisbon, Portugal).

APAP concentrations were quantified in the fungal culture supernatants by using a high-performance liquid chromatography system L-7100 (LaChrom HPLC System, Merck, Darmstadt, Germany) consisting of a quaternary gradient pump, and L-7400 UV detector. The whole system was controlled using the Merck HPLC System Manager software. The separation of the analytes was performed at ambient temperature in a Waters Spherisorb ODS2 column (5.0 µm, 4.6 × 250 mm) (Waters, Milford, MA, USA), using an isocratic condition with a mobile phase composed of water (pH 3.5, adjusted with orthophosphoric acid) and acetonitrile, at a 90:10 (*v*/*v*) and a flow rate of 1.0 mL/min. Detection was performed at 254 nm. Under these conditions, APAP, HQ, CAT, and APA could be separated within 12 min.

### 2.5. Cell Viability Assays

Cell viability assays were performed using the hepatocellular-carcinoma-derived HepG2 cell line (ATCC HB-8065) according to the method previously described [38]. Briefly, HepG2 cells were cultured in McCoy’s 5a modified medium supplemented with 10% heat-inactivated fetal bovine serum, 2 mM L-glutamine, 1% MEM nonessential amino acids, and 100 U/mL penicillin/streptomycin (Gibco, Life Technologies, Waltham, MA, USA), and maintained at 37 °C in a humidified incubator under 5% CO_2_. The cells were cultured in 24-well plates for 24 h to reach approximately 75% confluence. Then, depending on the experiments, the cultures were supplemented with different concentrations of APAP or with fungal samples (96 h of culture) from different initial concentrations of APAP supplemented with glucose (1.5 mg/mL), and finally with either control solutions containing 2.00 mM APAP or fungal samples with no glucose after 96 h (an initial APAP concentration of 2.00 mM and a final concentration less than 0.33 mM). After 48 h of incubation, 50 µL of PrestoBlue reagent^®^ (Thermo Fisher Scientific, Waltham, MA, USA) solution was added per 1 mL of culture medium and incubated for an additional time of 2 h. Plates were then analyzed for fluorescence emission in a Tecan Infinite M200 plate reader, using an excitation wavelength of 560 nm and an emission wavelength of 590 nm. The results were read using Tecan i-Control v. 1.4.5.0 plate reader software. Each experiment was performed using biological triplicates.

### 2.6. Transcriptome Analysis by Next-Generation Sequencing

Total RNA from selected *P. chrysogenum* var. *halophenolicum* cultures was isolated by phenolic extraction and mechanical homogenization. Briefly, the fungal mycelium from 2 mL of culture was harvested using centrifugation, resuspended in 800 µL of Trizol reagent (Thermo Fisher Scientific), and homogenized using agitation in a bed-beater for 2 min. After this process, 200 µL of chloroform was added to the samples and subsequently extracted using centrifugation. RNA was further purified and concentrated with column extraction using a Qiagen RNAeasy mini kit. The quality and concentration of the RNA samples were checked using UV spectroscopy in a Nanodrop 2000 microspectrophotometer (Thermo Fisher Scientific) and capillary electrophoresis in a Tape Station system (Agilent, Santa Clara, CA, USA). All the RNA extraction and further analysis were performed by using biological triplicates. The quality control results per sample are depicted in supplementary material (Figures S2 and S3). 

Transcriptomic analysis of the selected RNA samples was performed by Illumina next-generation sequencing in a NextSeq 500 system following the instructions from the manufacturer. Library preparation, sample barcoding, and pair-ended next-generation sequencing were carried out at the Genecore facility, EMBL, Heidelberg, Germany. Illumina sequencing reads were quality-filtered and trimmed with the Trimmomatic software [39] and aligned to the *Penicillium chrysogenum* genome (NCBI assembly ASM71027v1) with the STAR software [40] (Supplementary Table S1 and Figure S4). Normalization of the aligned reads, principal component analysis, and clustering and differential expression analysis between sample groups were carried out by DESeq2 and EdgeR algorithms embedded in the RNfuzzyApp suite (Supplementary Table S2) [41]. Annotation of the differentially expressed genes was based on the Biomart database [42]. Functional classification, gene ontology analysis, and the filtering of redundant terms were performed with the DAVID [43] and REVIGO applications [44]. BioCPR application was used for the calculation of correlation matrices and a heatmap representation [45].

Functional protein–protein interaction networks were retrieved from the STRING database considering a threshold of 0.75 in the combined prediction score [46]. Network clustering, enrichment, and comparative analysis were performed using the NetConfer application [47]. 

### 2.7. Statistical Analysis

Statistical analysis of cell viability data was performed by using Student’s *t*-test implemented in the GraphPad software. Gene expression data were statistically analyzed by the DESeq2 and EdgeR algorithms embedded in the RNfuzzyApp suite [41].

## 3. Results

### 3.1. Removal of APAP by P. chrysogenum var. halophenolicum and Cytotoxic Evaluation

**Figure 1:**
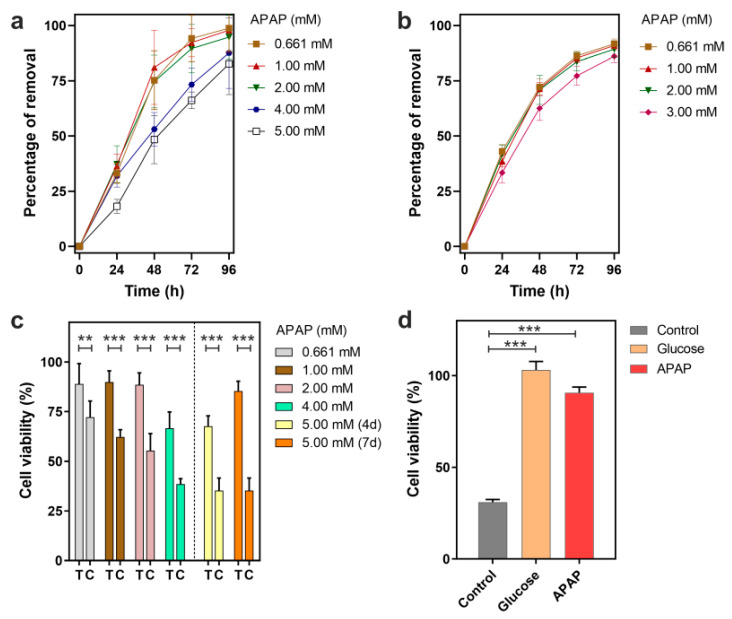
Removal of acetaminophen (APAP) by *P. chrysogenum* var. halophenolicum and determination of the toxic effect over HepG2 cell line. (**a**) Percentage of APAP removal by P. chrysogenum var. halophenolicum under cometabolism with glucose. (**b**) Percentage of APAP removal when used as the sole carbon source by the fungal strain. (**c**) Cell viability determination of the hepatoma HepG2 cell line when exposed to different APAP concentrations before (C) and after treatment with the fungal strain (T), and fungal cultures from cometabolism after 4 days (4 d) and 7 days (7 d). (**d**) Comparison of the effects of the bioremediation of APAP used as carbon source by P. chrysogenum var. halophenolicum quantified as the viability of HepG2 cell line: Control, 2.00 mM APAP; Glucose, fungal culture with an initial concentration of 1.5 mg/mL glucose; APAP, fungal culture with an initial concentration of 2.00 mM APAP. Statistical significance of the comparisons calculated with Student’s *t*-test was depicted by asterisks: *, *p*-value < 0.05; **, *p*-value < 0.001; ***, *p*-value < 0.0001.

To assess the APAP removal efficiency of *P. chrysogenum* var. *halophenolicum,* the fungal strain was cultivated in a mineral medium supplemented with different concentrations of APAP (0.661, 1.00, 2.00, 4.00, and 5.00 mM) and 1.5 mg/mL of glucose. The APAP levels were monitored with HPLC and the supernatant cytotoxicity was evaluated using the hepatoma cell line HepG2 model (Figure 1a,c). APAP toxicity against HepG2 cells was previously determined with a high-dose exposure experiment, showing a clear decrease in cell viability as a function of APAP concentration (Figure S1). In fact, a viability reduction in HepG2 cells of more than 25% was observed in 1.00 mM APAP, being more pronounced for APAP concentrations up to 2.00 mM. 

We observed that the presence of the fungus resulted in a decrease of 33.2%, 36.5%, 37.3%, 32.0%, and 18.2% of APAP after 24 h of culture at initial concentrations of 0.661, 1.00, 2.00, 4.00, and 5.00 mM, respectively (Figure 1a). APAP time course analysis demonstrated that after 96 h of culture, only levels of 1.2%, 2.1%, 5.2%, 12.4%, and 17.3% of APAP from an initial concentration of 0.661, 1.00, 2.00, 4.00, and 5.00 mM, respectively, were detected. 

Meanwhile, after 96 h of fungal treatment, a significant increase in HepG2 cell viability was found for all the concentrations tested (Figure 1c). The additional time of contact with *P. chrysogenum* var. *halophenolicum* in the cultures with higher levels of APAP (an initial concentration of 5.00 mM) also resulted in decreased toxicity. Therefore, experimental data highlight an unequivocally positive result of the fungal treatment over the detrimental effect of APAP on HepG2 cells. Moreover, we observed that cell death in HepG2 cells after fungal treatment was similar to the determined at the remaining APAP concentration in the media, indicating that cytotoxicity is mainly due to the presence of the drug.

According to the literature, there is only one publication reporting a *Penicillium* strain and its ability to use APAP (0.661 mM) as the sole carbon source, [30]. In the present study, we investigated the capacity of *P. chrysogenum* var. *halophenolicum* to use APAP at concentrations from 0.661 mM to 3.00 mM as the sole carbon source. The fungus was cultivated in mineral medium supplemented with 0.661, 1.00, 2.00, and 3.00 mM APAP. A control condition with 2.00 mM APAP and without the fungal strain was performed to investigate if the removal of APAP could be mediated by abiotic processes. In the control assay, no decrease in APAP was observed, which indicated that the removal of APAP using abiotic processes was negligible (data not shown). Compared to the 0.661 mM APAP, the percentage of removal for all the concentrations tested at different times presented the same trend (Figure 1b). The APAP concentration gradually decreased during 96 h of cultivation, being less pronounced after 48 h. Moreover, similar percentage values were obtained in the range from 0.661 to 2.00 mM APAP. For example, at 48 h of culture, 27.26%, 28.51%, and 29.03% of APAP from an initial concentration of 0.661, 1.00, and 2.00 mM, respectively, were observed. Since APAP abiotic reduction was not detected, these results indicated that the fungus not only could remove APAP when it is the sole carbon source, but the process is also independent of the initial APAP concentration at concentrations from 0.661 to 2.00 mM. It was reported that *Mucor hiemalis* tolerance to APAP increased at lower concentrations, because a higher uptake was obtained at 5 mg/L (50%), compared to 50 mg/L (8%). This ability to remove APAP was achieved in the first 24 h, remaining unchanged during seven days [29]. Meanwhile, at 96 h, *P. chrysogenum* var. *halophenolicum* was able to degrade more than 90% of APAP when the initial concentration was 1.00 mM (151.2 mg/L) (Figure 1b). 

The exposition to easily metabolizable carbon sources combined with specific xenobiotic compounds could affect the degradation efficiency of the last ones, due to different phenomena which include enzymatic competition and alterations in metabolic flux. In the present study, the APAP removal efficiencies in the presence of glucose were found to be slightly higher, or equivalent, for all the concentrations tested. In the case of 2.00 mM APAP, the removal efficiency after 4 days was 94.8 ± 1.1% and 89.3 ± 4.3%, with and without glucose, respectively.

Considering that 2.00 mM APAP was recently reported as the concentration from which severe growth arrest and little proliferation of HepG2 cells is observed in vitro [48], we further investigated APAP degradation with an initial concentration of 2.00 mM. It is known that the degradation of APAP in the environment often results in the accumulation of hydroquinone and p-aminophenol, metabolites that are toxic [31]. *P. chrysogenum* var. *halophenolicum* is transforming APAP to easily metabolizable substrates since under the experimental conditions not only hydroquinone and p-aminophenol were not detected but also cytotoxicity decreased. However, HepG2 cell viability was not 100% after fungal treatment, which could be hypothetically explained by dark-brown product(s) observed in APAP fungal cultures (Figure 1d). Furthermore, the dark-brown coloration was dependent on the initial concentration, presence of glucose, and time of incubation. A similar behavior was observed with Pseudomonas moorei KB4 during APAP degradation, where the brown colorization of cultures was detected with highest APAP concentration tested (50 mg/L) [49]. It has been reported that during APAP transformation, polymerization products of catechol or APA complexes could be formed and this accumulation leads to brown coloration [23,26]. Interestingly, we observed a delay in pigment formation on the batch with glucose, possibly due to the preferential use of the monomeric sugar instead of APAP. In fact, no glucose was detected after the first 24 h of culture (data not shown). 

### 3.2. Transcriptional Analysis of APAP Degradation

**Figure 2:**
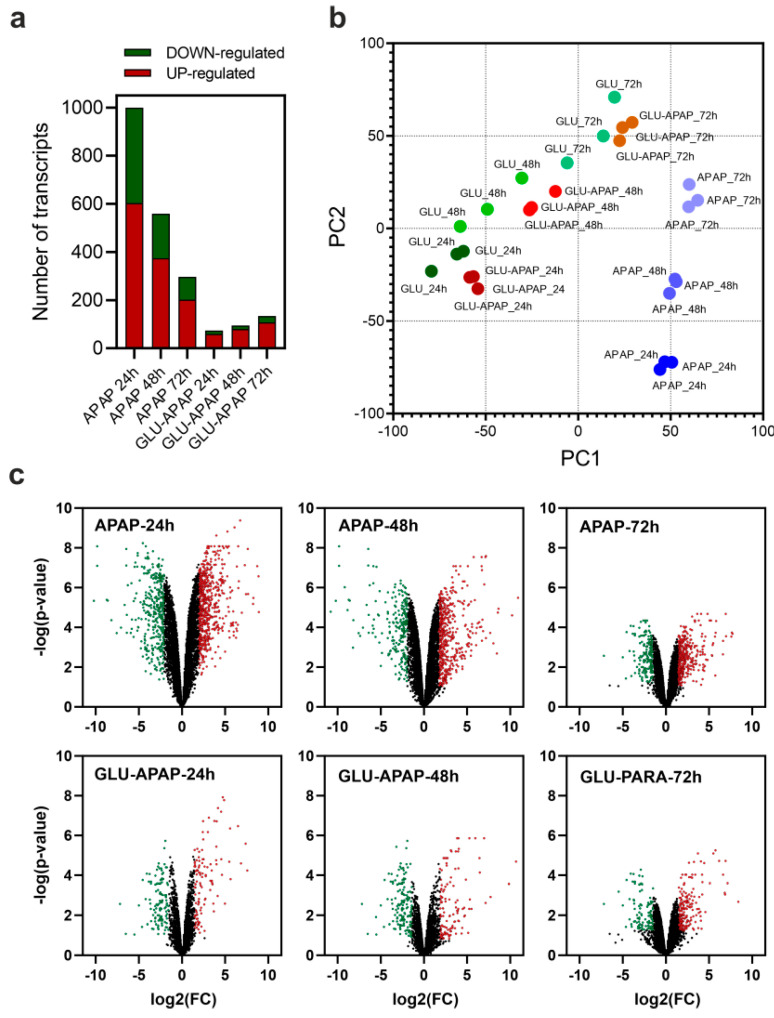
Transcriptomic analysis of *P. chrysogenum* var. *halophenolicum* during APAP degradation. (**a**) Number of differentially expressed mRNA transcripts at 24, 48, and 72 h of culture in media containing APAP or APAP and glucose as carbon sources refer to the cultures growth using only glucose. (**b**) Principal component analysis of gene expression in all the samples of the experiment using glucose (GLU), APAP (APAP), and glucose + APAP (GLU-APAP) as carbon sources. (**c**) Volcano plots of differentially expressed mRNA transcripts in presence of APAP or APAP and glucose, referred to the respective cultures growth in glucose as carbon source. Differentially expressed genes were considered when *p*-value < 0.05 and a −2.0 > logFC > 2.0, calculated by the EdgeR algorithm. In the volcano plots, upregulated gene transcripts are represented as red dots and downregulated transcripts by green dots.

Using transcriptomic analysis via next-generation sequencing, we studied the transcriptional pattern exhibited by the fungal strain during APAP degradation. The differentially expressed mRNA transcripts were determined by comparing samples where *P. chrysogenum* var. *halophenolicum* was grown in APAP with samples containing only glucose as the carbon source. Additional experiments were also performed by combining APAP and glucose as carbon sources. The APAP degradation either alone or together with glucose was followed during 72 h of culture in minimal medium, and the transcriptomic data were characterized every 24 h. The results are depicted in Figure 2.

Our results showed that the presence of APAP as the sole carbon source induces a visible transcriptional pattern in the fungal strain, characterized by a predominance of upregulated mRNA transcripts (Figure 2a). This pattern was especially evident in early culture times when the APAP was still present at high concentrations. Considering a p-value < 0.05 and a −2.0 > logFC > 2.0, at 24 h of culture, we detected 604 upregulated and 396 downregulated mRNA transcripts (Figure 2a,c). The number of differentially expressed mRNA transcripts decreased proportionally to the degree of APAP metabolization, reaching 202 upregulated and 95 downregulated transcripts at 72 h of culture (Figure 2a,c). Moreover, the raw expression data allowed a clear stratification of the sample groups and replicates, as determined using principal component analysis (Figure 2b). Curiously, the combination of APAP and glucose together abolished the dysregulation pattern observed in the presence of APAP as the sole carbon source (Figure 2a,c). The presence of an easily metabolizable source of carbon suggested the phenomenon of catabolic repression, often observed as the transition boundary between the primary and the secondary metabolism [50].

**Figure 3:**
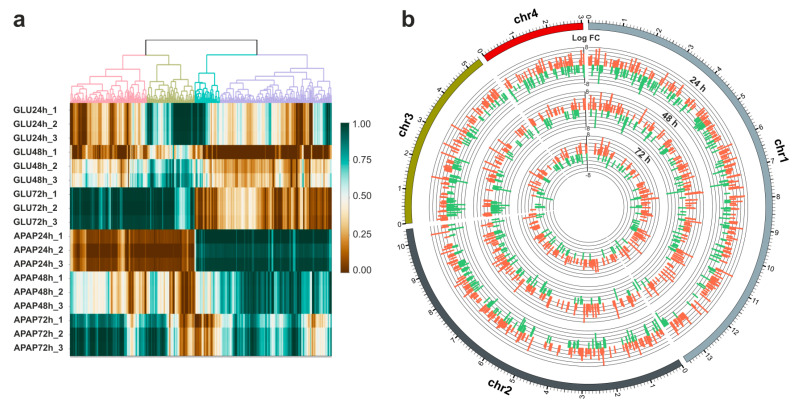
Spatial location and variance clustering of the differentially expressed transcripts during APAP degradation by *P. chrysogenum* var *halophenolicum*. (**a**) Variance clustering of the differentially expressed transcripts in presence of glucose or APAP as sole carbon sources, considering those with corrected *p*-value < 1 × 10^−5^. (**b**) Circular depiction of the chromosome map showing the relative position of the upregulated and downregulated transcripts, and the corresponding logFc values during APAP degradation by the fungal strain. The concentric circles represent the transcriptional pattern of gene expression at 24, 48, and 72 h of incubation with APAP, where the upregulated transcripts are represented in red bars and the downregulated mRNAs in green ones. Scale in chromosomes is represented as millions of base pairs.

The spatial mapping of differentially expressed genes within the *P. chrysogenum* genome showed a very interesting pattern of local events that are related to the culture time (Figure 3a). Each chromosome presented areas with a high density of upregulated units at 24 h of culture, that decreased with the metabolization of APAP, suggesting the existence of a metabolic induction phenomenon in specific genomic regions. Interestingly, at least four chromosomal territories that are highly transcriptionally upregulated at 24 h showed a transcriptional shutdown in the following culture times, accompanying the decrease in APAP levels in the culture supernatants. These regions cover chr1: ~12.5–13.5 Mb, chr2: ~6–7 Mb, chr3: ~4.5–5.5 Mb, and chr4: ~1.8–2.5 Mb. Moreover, the transcriptional dysregulation pattern induced by APAP also includes two clear regions in chr1 (~5–5.5 Mb) and chr3 (~0.2–1 Mb) enriched in downregulated genes, that at lower concentrations of APAP and longer culture times showed the opposite behavior (Figure 3b). This phenomenon is compatible with a transcriptional repression induced by higher concentrations of the drug. Variance analysis, using highly significant dysregulated mRNAs, clearly support the previous findings about the stratification of the different samples, and the dynamic transcriptional behavior in response to the exposure to APAP and the further metabolization (Figure 3a).

### 3.3. Functional Analysis of the Differentially Expressed Transcripts during APAP Degradation

**Figure 4:**
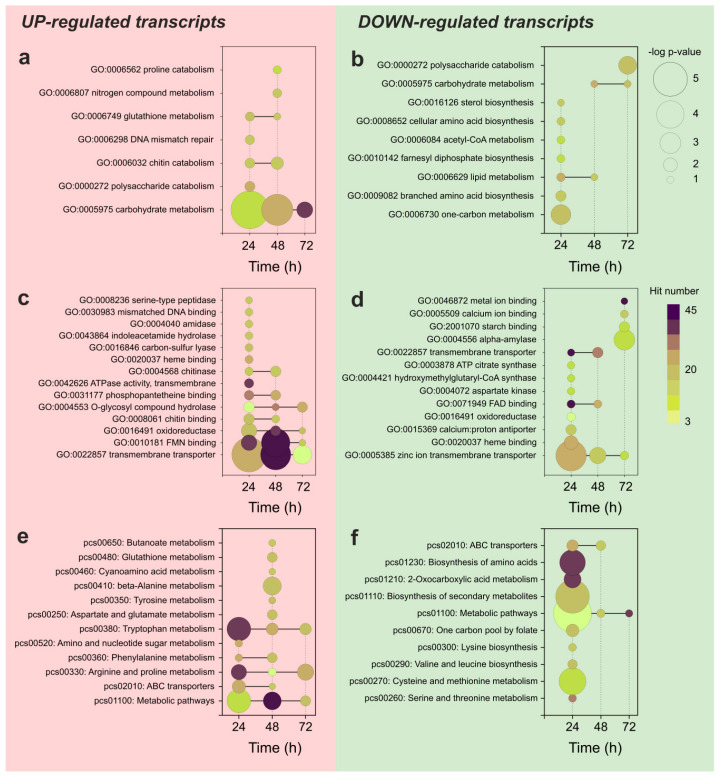
Functional analysis by gene set enrichment of the differentially expressed mRNA transcripts detected in cultures of *P. chrysogenum* var. *halophenolicum* containing APAP, performed by DAVID platform [43]. The left-hand side of the graph illustrates the enrichment analysis for the upregulated gene transcripts and the right-hand side is the analysis of the downregulated mRNAs. The size of the circular symbols depicting every functional category is proportional to the -log(p-value). The fill color of the symbols represents the number of functional hits detected within each category. (**a**,**b**) GO term enrichment analysis, biological process category; (**c**,**d**) GO term enrichment analysis, molecular function category; and (**e**,**f**) Pathway enrichment analysis, KEGG database.

Despite the accumulated knowledge of APAP degradation pathways in bacteria [21,23], the studies describing filamentous fungi as bioremediation agents able to degrade this drug are comparatively less extended [29,31]. Subsequently, the enzymes and degradation pathways used by filamentous fungi to degrade APAP are not well characterized. To give some insight into the possible metabolic pathways and individual proteins involved in APAP biodegradation, we performed a functional analysis based on gene set enrichment methods (Figure 4). 

Considering the upregulated gene transcripts, the GO enrichment analysis results showed an enrichment in genes related to the metabolism of high-molecular-weight polysaccharides represented by chitin, the DNA damage response and repair, and the catabolism of nitrogen compounds (Figure 4a,c). In more detail, and considering the up-regulated mRNA transcripts, the pathway enrichment analysis based on the KEGG database showed an overrepresentation of transcripts related to the metabolism of amino acids, glutathione, butanoate, and the transmembrane transport involving ABC transporters (Figure 4e). Interestingly, the complexity of the enrichment pattern decreased with the culture time and followd the APAP degradation. 

The downregulated transcripts did not show a clear enrichment pattern when analyzed by GO terms. Some terms related to divalent cation metabolism, binding, and transport across membranes were detected at 24 h of culture, but they were statistically less significant than those noticed in the upregulated transcriptome (Figure 4b,d). However, the pathway enrichment analysis allowed determining that at 24 h of culture, the downregulated transcripts were specifically related with the anabolism of nitrogen compounds, the biosynthesis of short-chain aliphatic amino acids, the metabolism of sulfur-containing amino acids, and the biosynthesis of secondary metabolites. In the downregulated transcriptome, the transition between culture times was more abrupt, and the enrichment pattern disappeared at 48 h of culture.

### 3.4. Protein–Protein Interaction Networks during APAP Degradation

**Figure 5:**
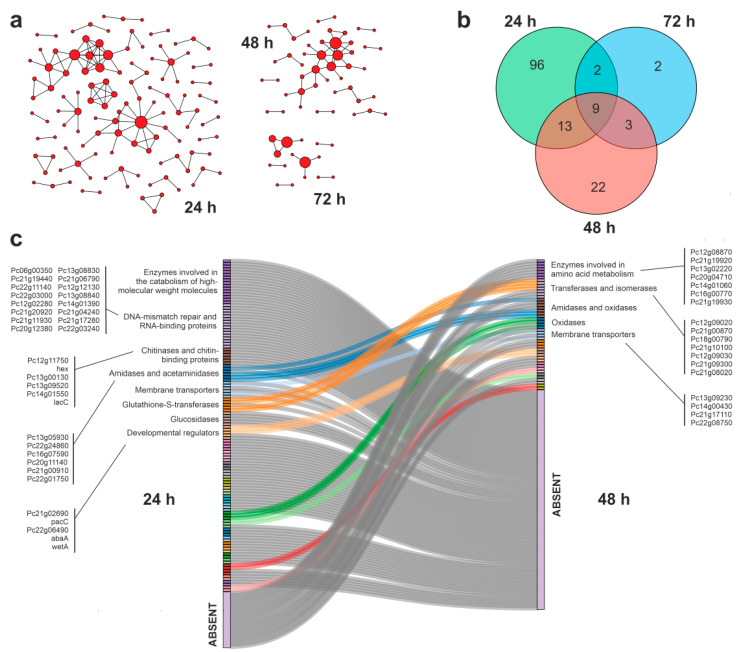
Analysis of the protein–protein interaction networks (PPIs) established among the proteins whose coding genes were overexpressed during APAP biodegradation by *P. chrysogenum* var. *halophenolicum*. The existence of the depicted PPIs was supported by the information deposited in the STRING database [46]. (**a**) schematic representation of the density of interaction nodes among the selected proteins at 24 h, 48 h, and 72 h of culture. (**b**) Venn diagram representing the number of proteins included in all the PPIs at the analyzed culture times. (**c**) Sankey plot showing the changes in community structure (densely connected groups of nodes) analyzed in the transition between 24 h and 48 h of culture performed by the Netconfer algorithm [47], illustrating the most relevant families of enzymes involved.

**Figure 6:**
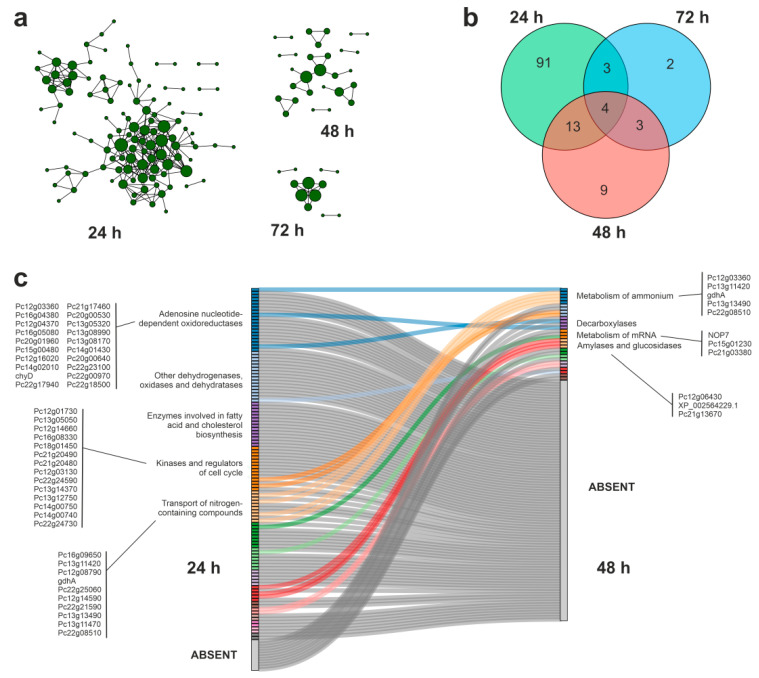
Analysis of the protein–protein interaction networks (PPIs) established among the proteins whose coding genes were downregulated during APAP biodegradation by *P. chrysogenum* var. *halophenolicum* and supported by the STRING database [46]. (**a**) schematic representation of the density of interaction nodes among the selected proteins at 24 h, 48 h, and 72 h of culture. (**b**) Venn diagram depicting the number of proteins included in all the PPIs at the analyzed culture times. (**c**) Sankey plot showing the changes in community structure (densely connected groups of nodes) analyzed in the transition between 24 h and 48 h of culture performed by the Netconfer algorithm [47], representing the most relevant families of proteins involved.

Fungal metabolism is often characterized by a great degree of flexibility, when compared with lower complexity microorganisms [51]. The selected *P. chrysogenum* strain has already shown an extraordinary metabolic potential, being characterized as an active metabolizer of phenolic compounds [32,52]. To gain a more detailed view of the metabolic events related with the degradation of APAP in minimal medium, we built the specific PPIs involving proteins encoded by up- and downregulated transcripts, taking advantage of the information deposited in the STRING database of protein–protein interactions [46]. The resulting interaction networks were compared at 24 and 48 h of culture, to determine the dynamic transitions between them as a response to the biodegradation of APAP (Figure 5 and Figure 6).

Considering the upregulated gene transcripts at 24 h of culture, 120 of the corresponding encoded proteins establish at least one functional interaction annotated in the STRING database (Figure 5a,b), corresponding to 19% of the total overexpressed coding transcripts. The APAP degradation by the fungal strain was accompanied by a decrease in the number of overexpressed gene transcripts whose coded proteins are involved in PPIs (47 at 48 h and 16 at 72 h). Interestingly, at 24 h, the PPIs clearly showed a stress-dependent pattern represented by the presence of a PPI cluster composed of proteins involved in DNA mismatch repair and RNA binding proteins, and two interacting clusters comprising enzymes involved in the catabolism of high-molecular-weight compounds and chitin. The PPIs also include a group of enzymes with amidase and acetaminidase activities, and a cluster formed by developmental regulators (Figure 5c). This complex PPIs pattern is substituted by a simpler and less dense network of interactions at 48 h of culture. Under these conditions, the APAP concentration has been reduced substantially, and the acute stress response disappeared. The community structure at 48 h is characterized by the maintenance of amidases, as well as by the presence of enzymes involved in the metabolism of amino acids and the appearance of the new PPI cluster composed of oxidases (Figure 5c).

The analysis of the PPIs established among the proteins whose genes were downregulated during APAP degradation showed a community transition pattern characterized by a dense network of interactions at 24 h of culture, that rapidly disappeared at 48 h (Figure 6a). At 24 h, the densest PPIs included oxidoreductases, dehydrogenases, ad nenzymes involved in fatty acid and cholesterol biosynthesis, and kinases involved in cell-cycle regulation. The transition between 24 and 48 h of culture was characterized by a dramatic decrease in the complexity in the community structure, where the oxidases were substituted by enzymes involved in mRNA metabolism, decarboxylases, and glucosidases. 

### 3.5. Putative Enzymes and Coding Genes Involved in APAP Degradation

**Figure 7:**
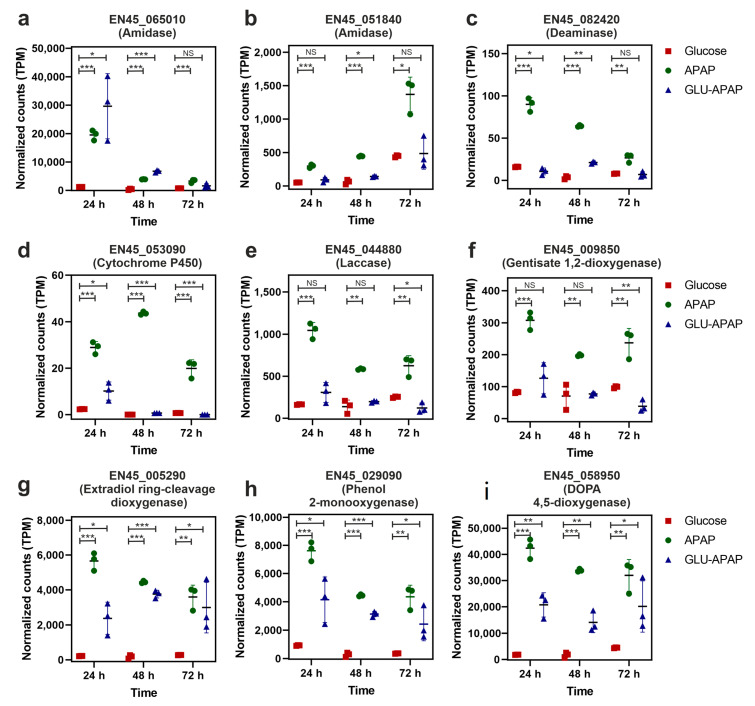
Normalized expression levels of selected gene transcripts encoding enzymes that are putative involved in the degradation of APAP by the strain of *P. chrysogenum*. Scattered dot plots represent the median value of expression and the standard deviation of the biological replicates. (**a**) amidase encoded by the EN45_065010 gene; (**b**) amidase encoded by the EN45_051840 gene; (**c**) deaminase encoded by the EN45_082420 gene; (**d**) cytochrome P450 encoded by the EN45_053090 gene; (**e**) laccase encoded by the EN45_044880 gene; (**f**) gentisate 1,2-dioxygenase encoded by the EN45_009850; (**g**) extradiol ring-cleavage dioxygenase encoded by the EN45_005290 gene; (**h**) phenol 2-monooxygenase encoded by the EN45_029090 gene; and (**i**) DOPA 4,5-dioxygenase encoded by the EN45_058950. Statistical significance of the comparisons determined by the EdgeR algorithm was depicted by asterisks: *, *p*-value < 0.05; **, *p*-value < 0.001; ***, *p*-value < 0.0001; NS, nonsignificant.

Considering the already-described pathways for APAP bioremediation by different microorganisms, we can conclude that at least three key families of enzymes should be involved: amidases, deaminases, and aromatic ring oxidases [24,26,49]. Taking advantage of the already-described transcriptomic data, we analyzed the expression time course of the mRNAs encoding enzymes belonging to the described families and induced by the presence of APAP in the culture media. The results of the analysis of the normalized gene expression are depicted in Figure 7. 

We detected three amidases encoded by genes EN45_065010, EN45_051840, and EN45_110750, whose transcripts were induced by the presence of APAP. The EN45_051840 gene transcript showed an early induction pattern favored by the presence of APAP, with higher expression levels at 24 h of culture (Figure 7a), whereas the EN45_051840 was more expressed at 72 h of culture (Figure 7b). Among the induced mRNA transcripts, we were able to detect an annotated transcript coding for a deaminase (EN45_082420) that also showed an early induction pattern which was more evident when the fungal strain was cultivated in the presence of APAP as the sole source of carbon (Figure 7c). 

The group of genes encoding aromatic ring oxidases induced by the presence of APAP was very diverse, including generic oxidoreductases as a P450-like cytochrome (EN45_053090, Figure 7d), a laccase (EN45_044880, Figure 7e), a phenol monooxygenase (EN45_029090, Figure 7h), two aromatic dioxygenases (EN45_009850, Figure 7f, and EN45_058950, Figure 7i), and a ring-cleavage dioxygenase (EN45_005290, Figure 7g). The general expression patterns of the corresponding coding genes during APAP degradation showed an early transcriptional induction at 24 h of culture, followed by a slow decrease in the mRNA levels at higher incubation times. Analyzing the normalized count levels, the mRNAs transcribed from the EN45_005290, EN45_029090, and EN45_058950 genes showed higher expression values, proportional to the remaining APAP concentration in the culture supernatants (Figure 7g–i).

**Figure 8:**
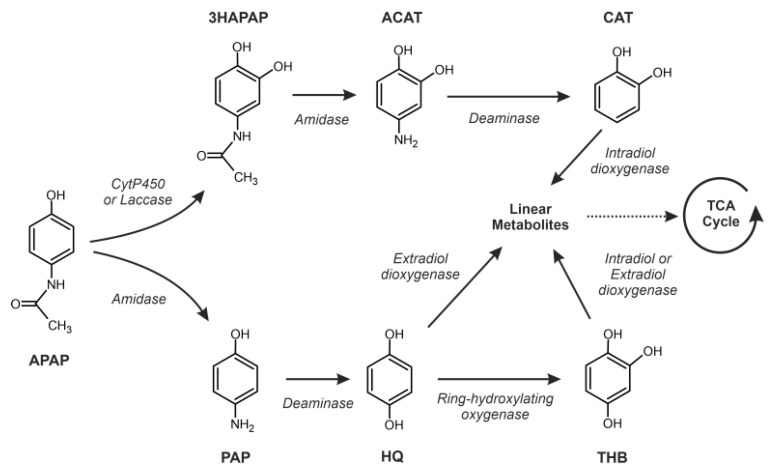
Proposed metabolic pathways for the degradation of APAP by *P. chrysogenum* var *halophenolicum* indicating the involved enzymes. APAP, acetaminophen; 3HAPAP, 3-hydroxyacetaminophen; ACAT, 4-aminocatechol; CAT, catechol; PAP, 4-aminophenol; HQ, hydroquinone; and THB, 1,3,4-trihydroxybenzene.

Considering the metabolic background of APAP degradation and the observed transcriptomic pattern, we proposed two possible pathways for the metabolization of APAP by *P. chrysogenum* var. *halophenolicum* (Figure 8). One of the proposed pathways could involve the transformation of APAP to HQ by deamidation and deamination, and a further oxidation by aromatic dioxygenases to generate linear compounds that should be introduced in the TCA cycle. The other pathway could produce CAT as a major APAP metabolite by coordinated hydroxylation, deamidation and deamination reactions (Figure 8). CAT would be likely oxidized by an intradiol-dioxygenase to release small linear compounds that would be used as a source of energy within the TCA cycle (Figure 8).

## 4. Discussion

In this study, we used a transcriptomic approach to illustrate the biodegradation of APAP by a *Penicillium* strain. Our results showed that either under cometabolism or as the sole carbon source, APAP can be efficiently removed by *P. chrysogenum* var. *halophenolicum.* Additionally, the APAP reduction was accompanied by a decrease in the corresponding toxicity of the culture supernatants against HepG2 cells (Figure 1). 

The degradation of APAP by other *Penicillium* strains has been previously demonstrated [30,53]. Hart and Orr (1975) reported the utilization of APAP for growth by *Penicillium* sp., when it was used as the sole carbon source. On the contrary, in *P*. *chrysogenum* var. *halophenolicum* cultures, the degradation of APAP without a net increase n fungal biomass was observed. At this point, we could speculate if the energy obtained from the APAP degradation could be used as maintenance, defined as the energy consumed for functions other than the production of new cell material. This maintenance component depends on relative growth rates, relative death rates, growth yield, and endogenous metabolism [54]. The flow of catabolism towards the maintenance of energy is a phenomenon often observed when a useful source of carbon and energy is present at a low concentration [55,56]. However, the APAP concentrations used in the present study were equal or higher than the concentration used by Hart and Orr (1975) [30]. These results prompted us to ask if APAP could be considered as a stress factor, and how carbon flux is channeled from APAP.

Model and clinical studies previously showed that the cellular toxicity of APAP is in part due to its DNA damage activity [57]. Our transcriptomic data revealed the induction of diverse genes encoding proteins related to the DNA damage response, especially at 24 h of culture. This acute transcriptional pattern in response to the presence of APAP is consistent with a stress effect, directly exerted over the fungal genome. Additional data that could support this idea were obtained from the analysis of the downregulated gene transcripts in the presence of APAP, that include a rich group of cell cycle regulators (Figure 5c). The inhibition of cell cycle transitions and the induction of apoptosis by APAP has been already described in eukaryotic cells [58]. 

Regarding the possible explanation of the carbon flow originated from APAP, the experimental data suggested that the drug is mainly metabolized by catabolic enzymes to produce energy. We obtained solid evidence that could support this hypothesis. Together with the “lack of growth” (i.e., cell viability is maintained relatively constant) of the fungal strain during APAP degradation, we also detected a visible induction of the expression of genes involved in the catabolism of high-molecular-weight compounds (Figure 5c). This overexpression was also accompanied by a downregulation of anabolic genes encoding enzymes involved in lipid and cholesterol biosynthesis (Figure 6c). The described results suggested that the carbon flux from APAP is mainly through the production of energy, that contributes to the maintenance of the cellular functions in a situation of a limited cell proliferation. In human cells, the anabolic suppression and decoupling of the energy metabolism induced by APAP has been also described, resulting in a limited cell proliferation by the suppression of the pathways required for cell growth [48].

Previous studies on the degradation of APAP by fungal strains postulated that APAP transformation involves a deacetylation catalyzed by an amidohydrolase or hydrolytic enzyme and subsequent aromatic ring fission [30,31,59], while the accumulation of 4-aminophenol (PAP) in the growth media was observed in the *Penicillium* cultures [30], in the *Scedosporium dehoogii* cultures, the APAP intermediates PAP and HQ were not found [31]. Recently, in the mixed culture with diclofenac, ibuprofen, naproxen, and ketoprofen, the 3-hydroxyacetaminophen was the metabolite detected from APAP transformation by a *Penicillium oxalicum* strain [53]. In the present study, we were not able to detect any of the cited intermediates. However, in the presence of APAP, we found three amidases (encoded by the genes EN45_065010, EN45_051840, and EN45_110750) with different patterns of expression in *P. chrysogenum* var. *halophenolicum* (Figure 7a,b and Figure 8). Since the first step of APAP deacetylation is performed by an amidase, cleaving the amide bond, we proposed the amidase (EN45_065010) that showed higher expression levels at 24 h, as presumably an aryl-acylamidase. In *Penicillium* species, the existence of an aryl-acylamidase and its function in the APAP degradation has been described before [30,60]. An alternative first step for APAP transformation could be its hydroxylation, as previously described in *Penicillium oxalicum* cultures [53]. In fact, the expression of the cytochrome P450 enzymes (encoded by EN45_053090) was observed in *P. chrysogenum* var. *halophenolicum*, particularly in the cultures with APAP (APAP and GLU-APAP) (Figure 7d and Figure 8). It is well known that cytochrome P450 enzymes play a central role in drug and xenobiotic detoxification, acting as terminal monooxygenases in several biochemical reactions such as hydroxylation and deamination [61]. *Aspergillus nidulans* required an amidase and a protein belonging to the cytochrome P450 superfamily for the utilization of benzamide [62]. Besides cytochrome P450 enzymes, laccases could also act on APAP, leading to 3-hydroxyacetaminophen formation [53]. A highly upregulated gene transcript encoding a laccase enzyme (encoded by the gene EN45_044880) was detected in *P. chrysogenum* var. *halophenolicum* in the presence of APAP at 24 h of culture (Figure 7e and Figure 8). Laccases are known to oxidize several phenolic substrates such as mono-, di-, polyphenols, methoxy-substituted phenols, aromatic compounds, and amines, including acrylamines or aminophenols [63].

According to the pathway proposed by Rios-Miguel et al. for APAP biodegradation [26], after amidase action, the second step could involve a deaminase. We also found a deaminase-domain-containing protein-encoding gene (EN45_082420) expressed in the fungal strain (Figure 7c and Figure 8). Therefore, it could be presumably involved in deaminating PAP that leads to HQ.

Hydroquinone, a frequently detected metabolite of APAP degradation, was not found in *P. chrysogenum* var. *halophenolicum* cultures. This result could be explained by its capability to use HQ as previously reported [35]. Therefore, it should be expected to find the specific hydroquinone 1,2-dioxygenase, an enzyme directly involved in HQ metabolization [64]. Similarly, as the work reported by Rios-Miguel et al. in bacteria [26], the fungal strain did not use a specific hydroquinone 1,2-dioxygenase. These authors suggested gentisate 1,2-dioxygenase instead, due to the chemical similarity of both compounds with two hydroxyl groups in para position [26]. In fact, a gentisate 1,2-dioxygenase (encoded by the gene EN45_009850) was identified (Figure 7f and Figure 8). However, gentisate 1,2-dioxygenase was expressed in the presence of APAP, GLU-APAP, and glucose as well, which makes the gentisate 1,2-dioxygenase (encoded by the gene EN45_009850) a weak candidate for ring cleavage. Despite the hydroquinone 1,2-dioxygenase not being identified in the fungal strain, a highly expressed extradiol ring-cleavage dioxygenase (encoded by the gene EN45_005290) on APAP conditions in comparison with GLU condition was observed (Figure 7g and Figure 8). On the other hand, a phenol 2-monooxygenase (encoded by the gene EN45_029090) and a Dopa 4,5-dioxygenase (encoded by the gene EN45_058950) were highly expressed in APAP cultures (APAP and GLU-APAP) compared with the glucose controls (Figure 7h,i and Figure 8). Therefore, an alternative route for HQ ring cleavage could be through the hydroxylation of HQ to originate 1,2,4-trihydroxybenzene [26].

Structurally identical to HQ, catechol (CAT) might be the last aromatic compound in the route of the 3-hydroxyacetaminophen (Figure 8). CAT degradation by *P. chrysogenum* var. *halophenolicum* has been previously demonstrated. Moreover, we reported that both dihydroxybenzenes, CAT and HQ, can be simultaneously removed by this fungal strain [34].

This work demonstrates that *P. chrysogenum* var. *halophenolicum* possess a versatile enzymatic system for the degradation of APAP. The degradation occurs without a significant increment in cellular mass and showed a transcriptomic pattern compatible with a stress response induced by the presence of APAP. The APAP biodegradation could be achieved using intracellular and extracellular enzymes, such as amidases, CYP450, laccases, and extradiol dioxygenases, among others, making *P. chrysogenum* var. *halophenolicum* an excellent candidate for APAP remediation. 

## Data Availability

Next-generation sequencing raw data have been deposited into the Sequence Read Archive (SRA) repository under the Bioproject ID: PRJNA943349.

## Supplementary Materials

The following supporting information can be downloaded at: https://www.mdpi.com/article/10.3390/jof9040408/s1, Supplementary Table S1: alignment statistics for RNAseq reads in all the selected samples. Supplementary Table S2: differentially expressed gene transcripts in all the studied comparisons, including functional annotations. Figure S1: Cell viability experiments in the human cell line HepG2 exposed to different acetaminophen (APAP) concentrations during 24, 48, 72 and 96 h. Figure S2: analysis of the total RNA samples extracted from *Penicillium chrysogenum* var *halophenolicum* employing a Tape Station system, and cultured under different conditions, as described in the material and methods section. Figure S3: RNA profiling by capillary electrophoresis from *Penicillium chrysogenum* var *halophenolicum* of the selected RNA samples. Figure S4: Next-generation sequencing count distribution in all the analyzed samples before normalization.
