# Supplementary material for: Transcriptomic Analysis of Acetaminophen Biodegradation by Penicillium chrysogenum var. halophenolicum and Insights into Energy and Stress Response Pathways
Source: J Fungi (Basel). 2023 Mar 27;9(4):408. doi: 10.3390/jof9040408 (PMC10146002; doi:10.3390/jof9040408)
Supplement: Supplementary file 1 [file jof-09-00408-s001.zip › Suppl Figures Revision 1.pdf]

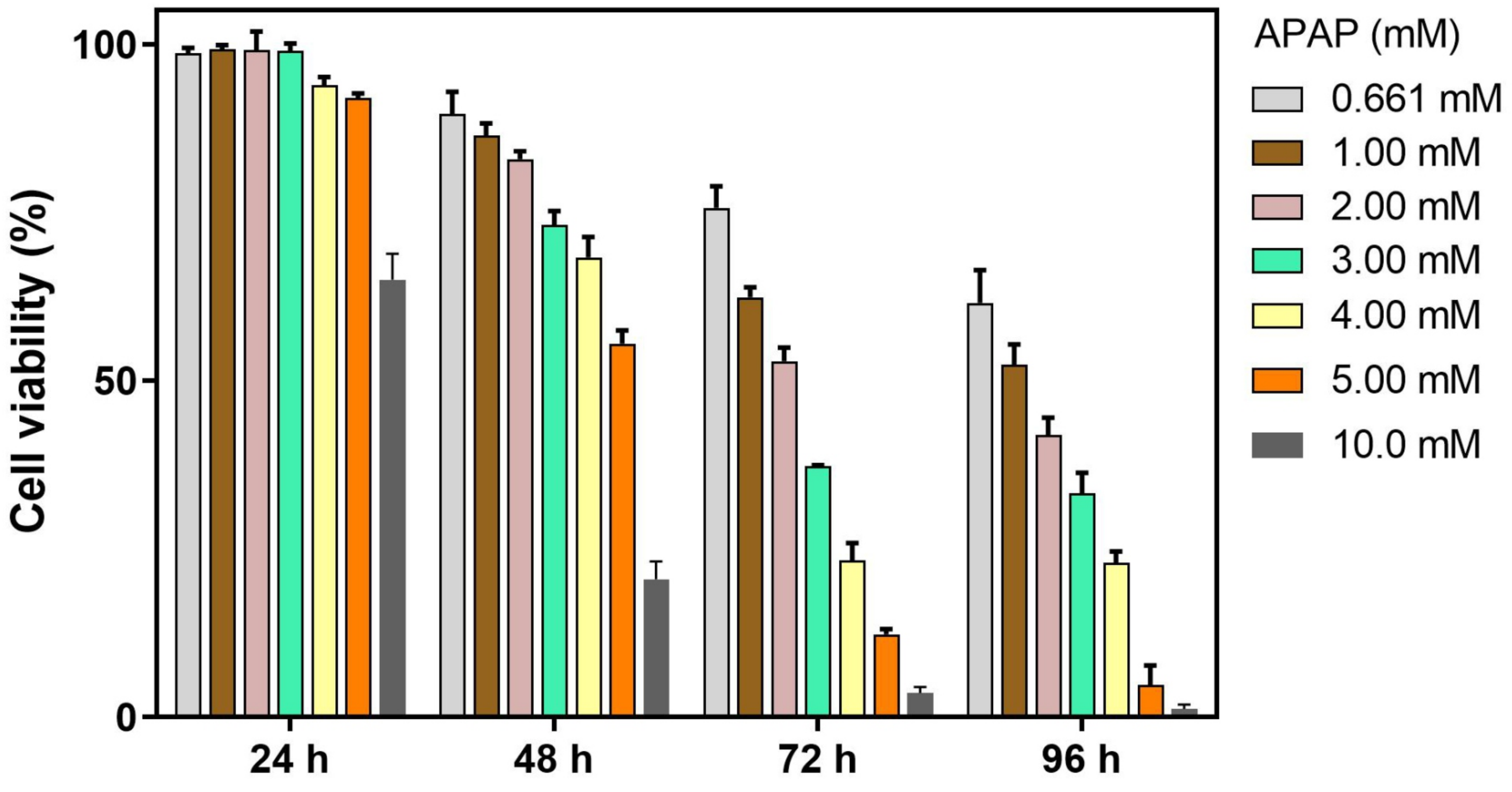

Figure S1: Cell viability experiments in the human cell line HepG2 exposed to different acetaminophen (APAP) concentrations during 24, 48, 72 and 96 hours. Cell viability was quantified by the PrestoBlue reagent as described in the Material and Methods section. The values of cell viability represented in the figure are relative to the controls without APAP. The bars depict the standard deviation among 5 independent biological replicates.

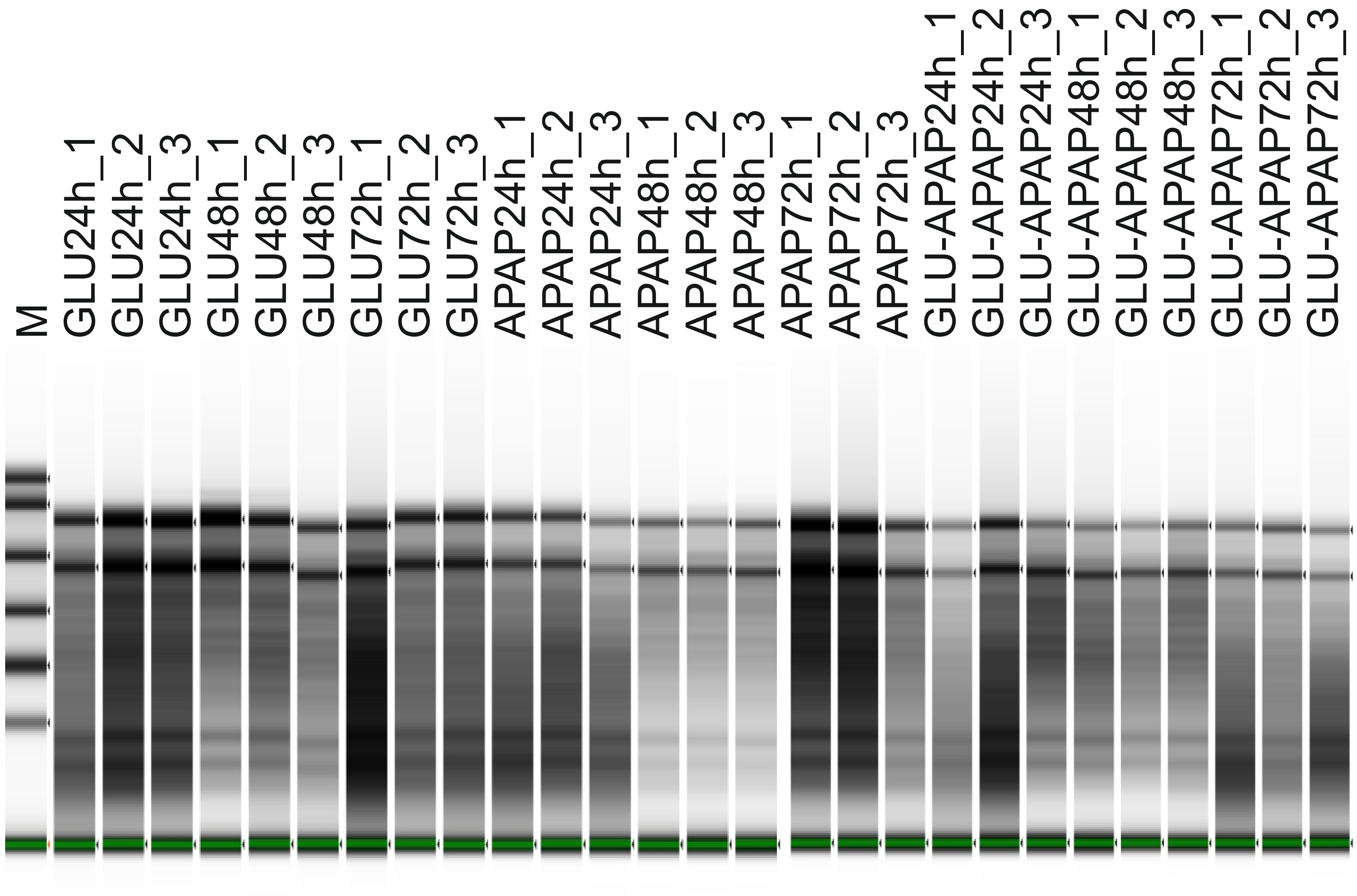

Figure S2: analysis of the total RNA samples extracted from *Penicillium chrysogenum* var *halophenolicum* employing a TapeStation system, and cultured under different conditions, as described in the material and methods section. GLU, fungal strain cultured with glucose as a sole carbon source; APAP, fungal strain cultured in the presence of acetaminophen as carbon source; and GLU-APAP, fungal strain cultured in the presence of glucose and acetaminophen as carbon sources.

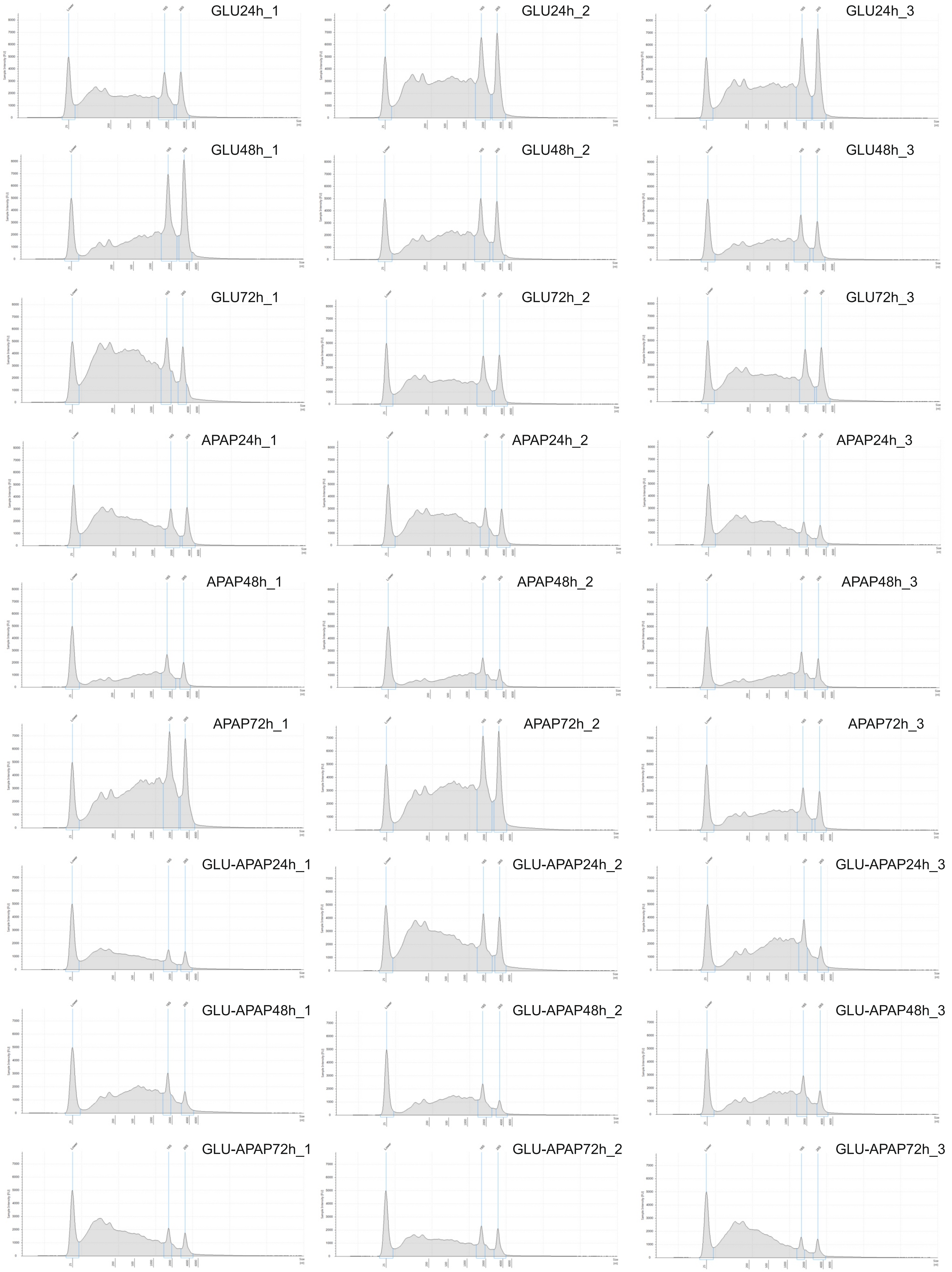

Figure S3: RNA profiling by capillary electrophoresis from *Penicillium chrysogenum* var *halophenolicum* employing a TapeStation system, and cultured under different conditions, as described in the material and methods section. GLU, fungal strain cultured with glucose as a sole carbon source; APAP, fungal strain cultured in the presence of acetaminophen as carbon source; and GLU-APAP, fungal strain cultured in the presence of glucose and acetaminophen as carbon sources.

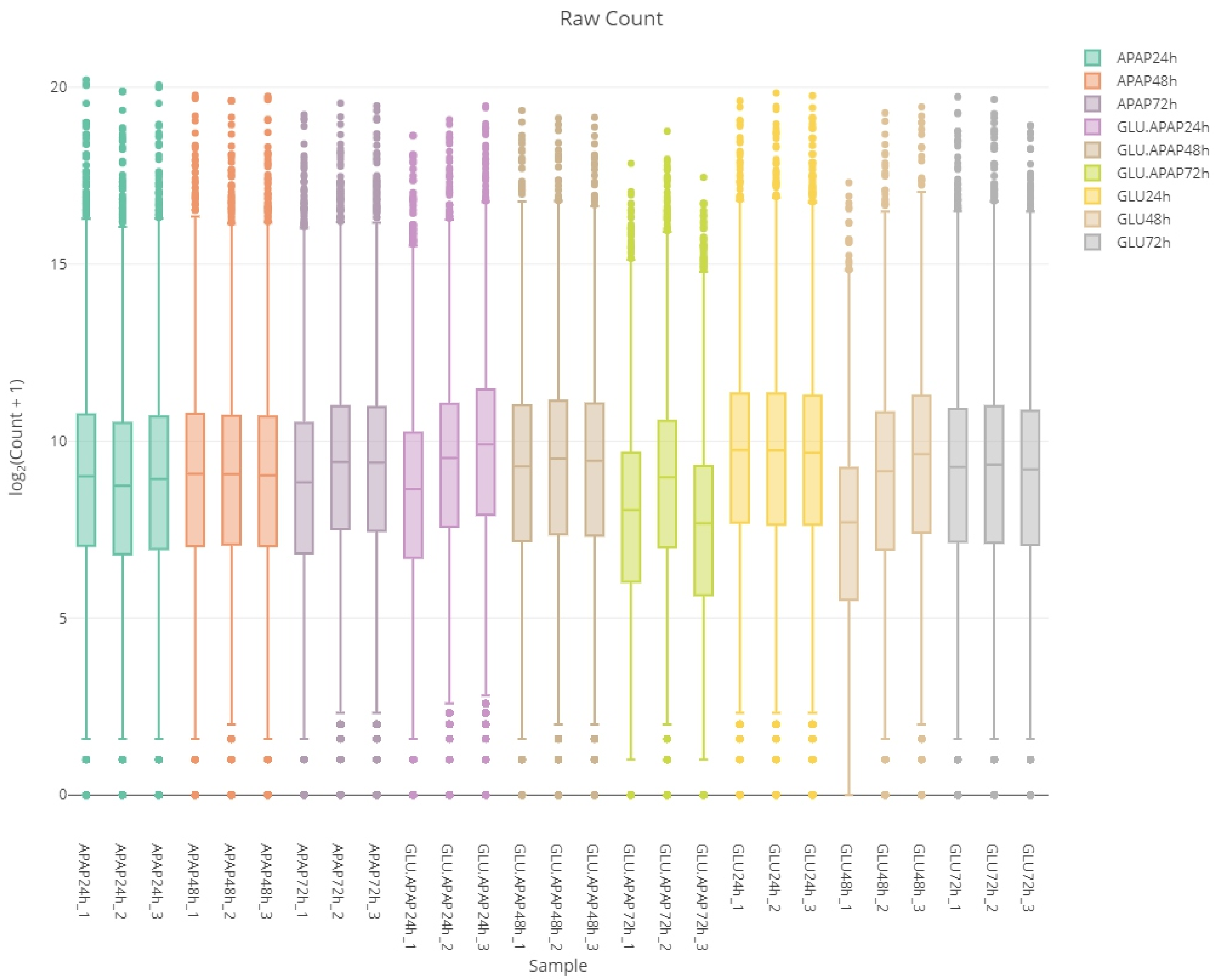

Figure S4: next generation sequencing count distribution in all the analyzed samples before normalization.
